# A giant solitary fibrous tumor of the adrenal gland in a 13-year old: a case report and review of the literature

**DOI:** 10.1186/s13256-019-2163-z

**Published:** 2019-08-08

**Authors:** Hailu Wondimu Gebresellassie, Yusuf Mohammed, Brahenu Kotiso, Bereket Amare, Aemero Kebede

**Affiliations:** 1 Addis Ababa University, College of Health Sciences, School of Medicine, Addis Ababa, Ethiopia; 2 Ethiotebib Hospital, Addis Ababa, Ethiopia; 3 Department of Pathology, Addis Ababa University, College of Health Sciences, School of Medicine, Addis Ababa, Ethiopia; 4 Dr Aemiro Higher Clinic, Addis Ababa, Ethiopia

**Keywords:** Solitary, Fibrous, Tumor, Adrenal, Gland

## Abstract

**Introduction:**

Solitary fibrous tumors are tumors of mesenchymal origin that occur in the extremities and occasionally in pleura, meninges, and so on, but are extremely rare in the adrenal gland. Their biological behavior is variable but mostly benign.

**Case presentation:**

A 13-year-old Oromo girl presented with a progressively increasing right upper abdominal mass of 3 years’ duration. She had dull dragging pain and an occasional low-grade fever. On examination she had 20 × 20 cm mass with well-defined medial and inferior border. Both ultrasound and computed tomography scan showed a highly vascularized mass arising from her right adrenal gland but she had neither the constitutional symptoms of a functional adrenal tumor nor an abnormal biochemical test. Surgical resection showed a vascularized mass with attachments to the right lobe of the liver with a weight of 1900 g. It was found to be a giant solitary fibrous tumor of her right adrenal gland with infrequent mitosis. She stayed for 5 days after surgery and was discharged. She showed remarkable recovery at follow-up at 3 months.

**Conclusion:**

Although very rare, solitary fibrous tumor of the adrenal gland should be considered in differential diagnosis of adrenal masses.

## Introduction

Solitary fibrous tumors (SFTs) are tumors of mesenchymal origin occurring mainly in the extremities but also in the pleura, meninges, and so on [1] . SFTs are extremely rare in the adrenal gland with only six or seven case reports to date [2]. SFTs have variable biological behavior but are benign most of the time with rare recurrence and metastasis. Bland spindle cell proliferation with “patternless” architecture and staghorn vessels are seen in histological examination [2].

The presence of nuclear atypia, hypercellularity, greater than 4 mitosis/10 high-power field (HPF), and necrosis may be associated with, but not predictive of, aggressive clinical behavior. The current treatment is complete surgical resection [2, 3].

## Case presentation

A 13-year-old Oromo girl presented with a progressively increasing right-sided abdominal mass, low-grade intermittent fever, and a dull right upper abdominal pain of 3 years’ duration with no other associated symptoms. There were no known past illnesses and there was no family history of similar illness. She was given pain medications and antibiotics on various occasions but there was no improvement.

Her general appearance was not acutely sick looking. Her vital signs were within normal limits. The pertinent abnormal finding was right-sided abdominal mass with well-defined medial and inferior border extending to right subcostal region. Complete blood count (CBC), urine analysis, and organ function tests were all normal. Ultrasound and a computed tomography (CT) scan demonstrated a huge vascular suprarenal mass displacing her right kidney caudally; it measurements were 16 × 19 cm and it contained multiple internal calcifications. There were no enlarged regional nodes and no vascular invasion.

Laboratory tests for functional adrenal tumors including serum and 24-hour urine metanephrines were all normal.

A working diagnosis of huge nonfunctional adrenal tumor was made and our patient underwent exploratory surgery through a bilateral subcostal incision. The operative findings were a well-capsulated and highly vascularized mass arising from the superior aspect of her right kidney, which got a significant blood supply from the right lobe of the liver. The mass was successfully and completely resected and the specimen subjected to histopathology.

The histopathology report showed 18 × 15 × 12 cm white solid mass with necrotic center arising from right adrenal gland. There was a patternless proliferation of spindle cells and ovoid cells that had mild pleomorphic nuclei and focally hyalinized stroma containing blood vessels. Mitosis was seen infrequently. These findings were consistent with a SFT of the adrenal gland.

Our patient was followed-up for 3 months and is doing remarkable well and members of her family were very grateful.

**Fig. 1:**
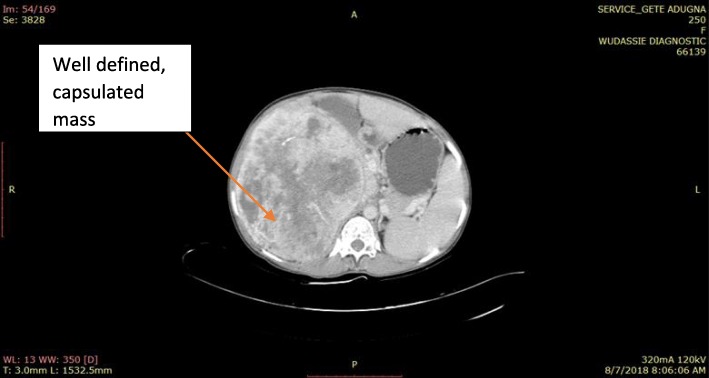
Computed tomography scan of solitary fibrous tumor of right adrenal gland of a 13-year-old girl

**Fig. 2:**
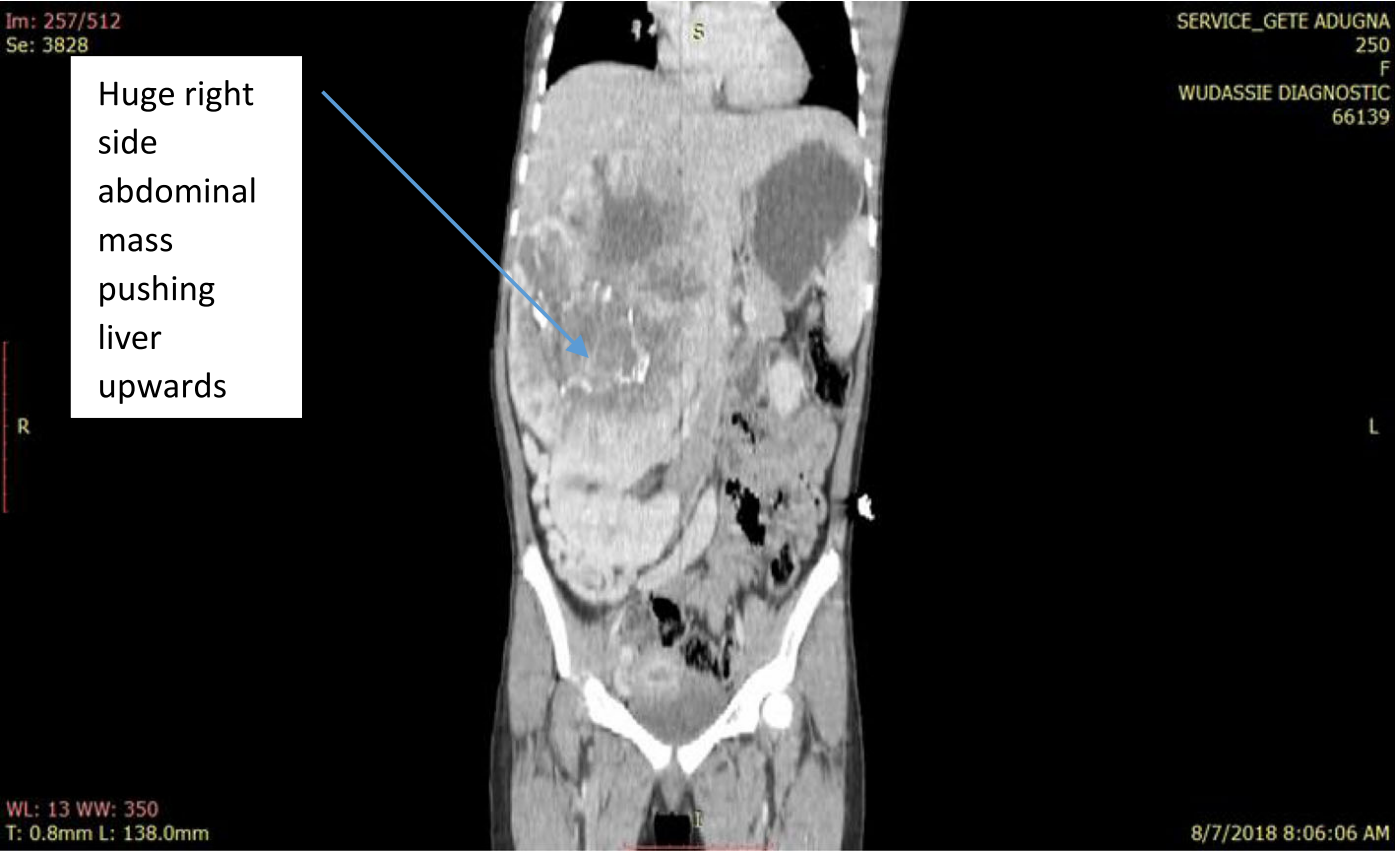
Computed tomography scan of solitary fibrous tumor of right adrenal in 13-year-old girl

**Fig. 3:**
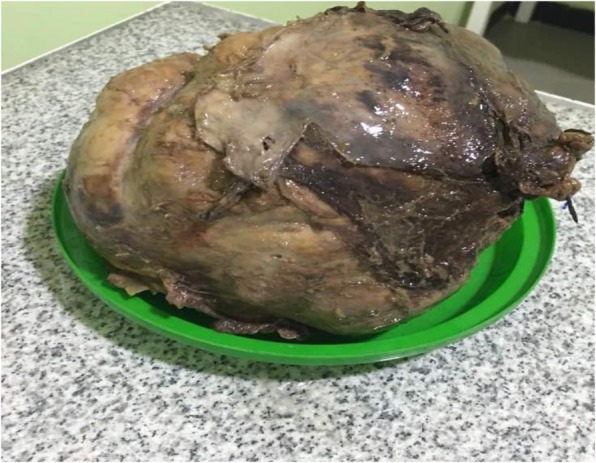
Resected specimen of solitary fibrous tumor after it was treated with formalin for histopathology

**Fig. 4:**
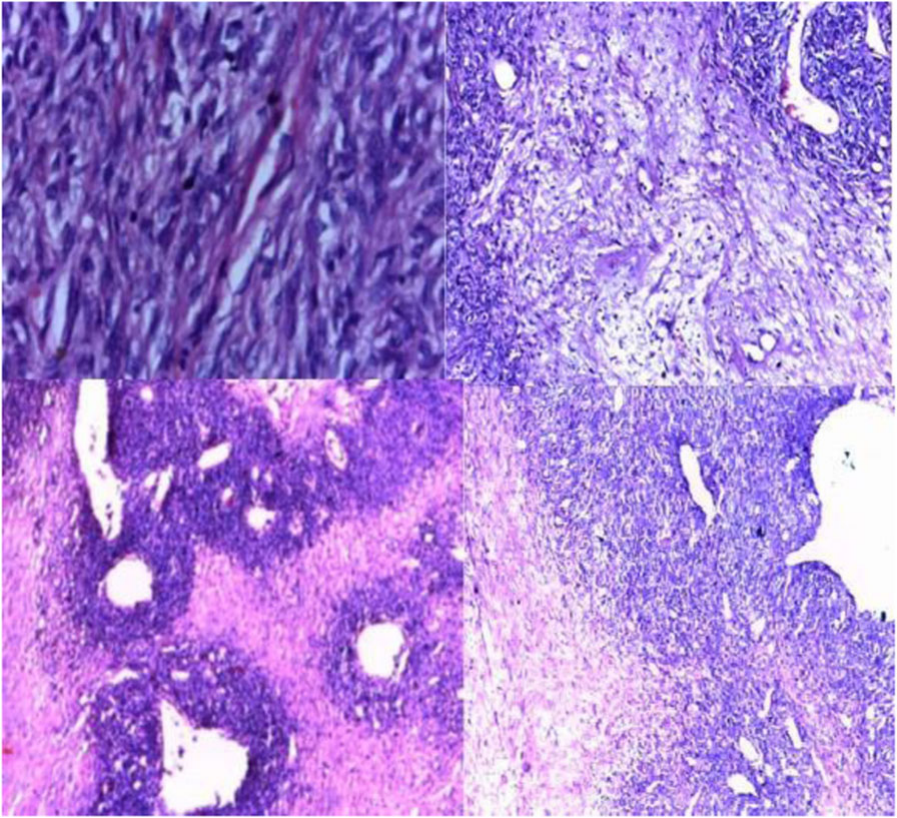
Histologic slides showing a patternless proliferation of spindle cells and ovoid cells that had mild pleomorphic nuclei and focally hyalinized stroma containing blood vessels. Mitosis seen infrequently

This is the sixth or seventh case of its kind in the world to the best of our knowledge (Figs. 1, 2, 3, and 4).

## Discussion

This patient presented with a huge (18 × 15 × 12 cm) slow-growing right adrenal mass with no symptoms of a functional adrenal tumor. Adrenal cortical tumors are common but tumors greater than 20 cm in diameter are unusual. A larger nonfunctional adrenal mass measuring 32 cm in diameter with a weight of 1700 gram was reported by Li and colleagues from Department of Oncosurgery at Anyang Tumor Hospital, Henan, China [4].

There was a report by Treglia *et al.* on a patient who underwent investigation for fever of unknown origin and was later found to have SFT of adrenal gland [5]. Our patient also had a low-grade intermittent fever but it was not objectively measured.

The intraoperative finding of a very highly vascularized mass and histopathology finding of patternless proliferation of spindle cells fit with the expected finding of SFT [2].

SFTs have a variable biological behavior but are mostly benign. A long history and histopathology report of infrequent mitosis as well as absence of invasion or metastasis on imaging and surgery suggest that it belongs to the benign end of the spectrum in SFT [1, 5]. A positive stain for CD34, BCL2, STAT6, and CD99 are neither specific nor sensitive but are useful [3].

Complete surgical excision is the recommended treatment in SFT and that was done for our patient [2].

SFT arising in an endocrine gland in general or adrenal gland in particular is rare and a Medline® search showed only a few case reports [6–8]. All the reports were in adults (the youngest being 23 years of age and the oldest 71 years of age) unlike our patient who was just 13 years.

Most of the reported cases of SFTs of the adrenal gland were discovered incidentally. Toniato *et al*. reported a very rare bilateral SFT of the adrenal gland in a 54-year-old patient with a bilateral adrenal incidentaloma [2]. Prevot *et al.* reported SFT in 42 years old women discovered accidentally on imaging and the mass remained unchanged for 5 years before the she agreed for surgery [9]. Bongiovanni *et al*. reported a SFT incidentally discovered in the left adrenal gland of a 22 weeks pregnant 23-year-old woman [10]. The mass was laparoscopically resected after she gave birth via spontaneous vaginal delivery [10]. Ho *et al*. also reported SFT in a 71-year-old Arab man [10, 11].

Our patient is not only the youngest but also had the biggest SFT ever reported. This is the sixth or seventh case of its kind in the world to the best of our knowledge.

## Conclusion

Although rare, SFT should be considered in the differential diagnosis of nonfunctional adrenal mass.

## Data Availability

No special software was used in this study except Mendeley for reference citing. CT scan film is available from the author and pathology specimen is available in histopathology department of our hospital.
